# Development of a Culturally Tailored Text Message Maternal Health Program: TextMATCH

**DOI:** 10.2196/mhealth.7205

**Published:** 2017-04-20

**Authors:** Rosie Dobson, Robyn Whittaker, Hannah Bartley, Augusta Connor, Ruyan Chen, Mairead Ross, Judith McCool

**Affiliations:** ^1^ National Institute for Health Innovation School of Population Health University of Auckland Auckland New Zealand; ^2^ Waitemata District Health Board Auckland New Zealand; ^3^ Epidemiology and Biostatistics School of Population Health University of Auckland Auckland New Zealand

**Keywords:** mHealth, short message service, maternal health, culture, text messages

## Abstract

**Background:**

Mobile phones are increasingly being used to deliver health information and health services globally. Mobile health (mHealth) interventions may be well-suited for minority groups with greater barriers to accessing traditional health services. However, little has been written about the process of culturally adapting interventions for multiple ethnic and cultural minorities within a population.

**Objective:**

This study describes the process of developing a culturally tailored text message-based maternal health program (TextMATCH: Text for MATernal and Child Health) for Māori, Pacific, Asian, and South Asian families living in New Zealand. We report on engagement and acceptability of the TextMATCH program.

**Methods:**

Program data was examined to describe engagement with the program 18 months after implementation. Telephone interviews were conducted with a sample of participants who consented to provide feedback on acceptability and relevance of the program.

**Results:**

A total of 1404 participants enrolled in TextMATCH over 18 months, with 18.52% (260) actively opting out at some point (after 0 to 17 months of messages). It was found that 356 (70.9%) of the 502 eligible participants actively switched from the initial pregnancy program to the baby program after delivery. Phone interviews were conducted with 29 participants including 6 who had withdrawn (duration of program from 3 to 16 months). Only 2 participants reported that the program was not useful, with the remainder rating the usefulness of messages positively (average 4.24 out of 5). All participants stated that the messages were relevant, culturally appropriate, and easy to understand. Most were happy with the specific advice and the language options provided.

**Conclusions:**

We have demonstrated the importance of an intensive approach to the development of a culturally adapted and tailored mHealth program for multiple different cultural minority groups within our population.

## Introduction

### Background

Mobile health (mHealth) is the use of mobile devices, including mobile phones, to deliver health services and information [1]. Due to the high use of mobile phones and the low cost of delivery, this mode of communication appears as an ideal platform for the delivery of health interventions. mHealth interventions are often designed as a means to reach underserved groups like ethnic or cultural minorities. This is justified due to the following reasons: (1) traditional health promotion methods often fail to serve the group in question; (2) the low cost of mHealth interventions make mHealth interventions feasible in low-resource environments; (3) mobile phone use or use of specific capabilities like text messaging or voice calling are common within the group targeted; and (4) mHealth has proven acceptable or effective in other, similar areas, or in feasibility studies [2-8].

Adapting effective mHealth programs for multiple minority populations requires attention to relevant cultural characteristics [2-4,6,9,10]. Cultural adaptation may reflect the nuances of language and cultural practices, so that health messages and services reflect their history, health beliefs and norms, social practices, and political and economic characteristics [10-14].

Some aspects of cultural adaptation that may improve the sustainability and effectiveness of mHealth include the consideration of users’ health beliefs and health literacy levels in message development [2,5], and the accommodation of local language and preferences in message content [3]. Cultural adaptation of message content in mHealth interventions has been executed in varying degrees from basic language translation to a sophisticated process of consultation with prospective users and data collection on barriers to services and health.

The process of the cultural adaption of mHealth interventions can be broadly categorized into three categories:

1. Translation only: mHealth intervention where content has been translated into the local language or a language nominated by users.
2. Translation and addressing group-specific barriers: the use of a local or nominated language, alongside content tailored to barriers in accessing services or health information which were faced by the cultural group concerned.
3. Translation and sophisticated adaptation: the use of a local or nominated language, alongside more complex adaptation and development processes, involving members of the target group.

However, there is a paucity of evidence to support the effectiveness and desirability of cultural adaptation practices in mHealth. In instances where perceptions of culturally adapted mHealth interventions have been described, these perceptions are positive [3,4,6,15-18]. A common factor is the lack of investigation of the cultural appropriateness of messages specifically.

One area of mHealth with some published descriptions of cultural adaptation processes is in the field of maternal health. There is growing support for the use of mHealth, including text messaging, in the field of maternal health [19], particularly for the advancement of the millennium development goals to reduce child mortality and improve maternal health in certain populations.

Text4baby in the United States is a free maternal health text message program, which is delivered in English and Spanish, for pregnant women and mothers of babies aged under 1 year. A survey assessed the linguistic appropriateness of the Spanish messages finding that 98.3% of respondents receiving the Spanish messages found the content to be clear and understandable [18]. In addition, Parker et al [20] detailed the translation, back translation, and intelligibility testing of English text messages from the US-based Text4baby intervention, translated into Russian. Expert checks of the messages were used to test the intelligibility of the messages, which informed pregnant women and mothers on how to keep themselves and their baby healthy, according to gestational age [20].

Mobile Alliance for Maternal Action (MAMA) has conducted cultural adaptation of a generic set of MAMA text messages to create new versions including ChatSalud and Mom Connect [21,22]. In Nicaragua, ChatSalud delivered sexual and reproductive health-oriented information to reduce teen pregnancy and the infant and maternal mortality associated with it [21]. In-depth interviews with adolescents who had participated in a pilot study of ChatSalud contributed relevant information about their particular barriers to sexual and reproductive health. Local health experts were also consulted about development of message content, given their experience of the population health threats in the area [21]. In South Africa, Mom Connect text messages collected data from the mother during pregnancy and the first year of the child’s life, and delivered age- and stage-specific messages to inform and support pregnant women and mothers [22]. Focus groups with prospective users and consultation with local health experts sought to ensure that the barriers addressed were relevant to the target group. These included intimate partner violence, depression, and persuading partners to use condoms during pregnancy to prevent human immunodeficiency virus (HIV) transmission. These processes resulted in MAMA South Africa message content being provided in six different languages and incorporating user comments and stories [22].

Although these studies have shown the feasibility of cultural adaptation of text messaging programs in maternal health, more evaluation of such interventions is needed to determine their effectiveness [20].

In New Zealand, we have developed TextMATCH (Text for MATernal and Child Health), an educational and supportive text message program for pregnant, new mothers (of infants aged until 2 years), and other family members. This program was funded by the New Zealand Ministry of Health as part of a broader initiative (*Healthy Babies Healthy Futures, HBHF*) targeting the increasing rates of childhood obesity (11% of children aged 2-14 years in New Zealand are reported to be obese) [23]. Targeting interventions to families is seen as vital in addressing increasing obesity rates. Commencing in pregnancy, maternal and child nutrition and physical activity influence physiology in ways that contribute to disease risk and lay the path for lifelong health.

TextMATCH was designed to cover three specific areas:

- improve women’s health during pregnancy and the postnatal period, through promotion of healthy eating and physical activity;
- promote healthy feeding of babies including encouraging and supporting breastfeeding; and
- promote healthy feeding (including the introduction of healthy first foods) and physical activity of children at preschool age.

TextMATCH, and the broader HBHF initiative it is embedded in, have been developed specifically for Māori (the indigenous population of New Zealand), Pacific, Asian, and South Asian families. These are minority groups with particularly high rates of childhood obesity (Māori 19% and Pacific 27%) [23] or with particular needs arising from being new migrants who may not be well-connected to, or have experience with, the New Zealand health system. This paper describes the development and cultural adaptation of TextMATCH specifically for these groups and the feedback on its acceptability to date.

### Intervention Development

**Figure 1:**
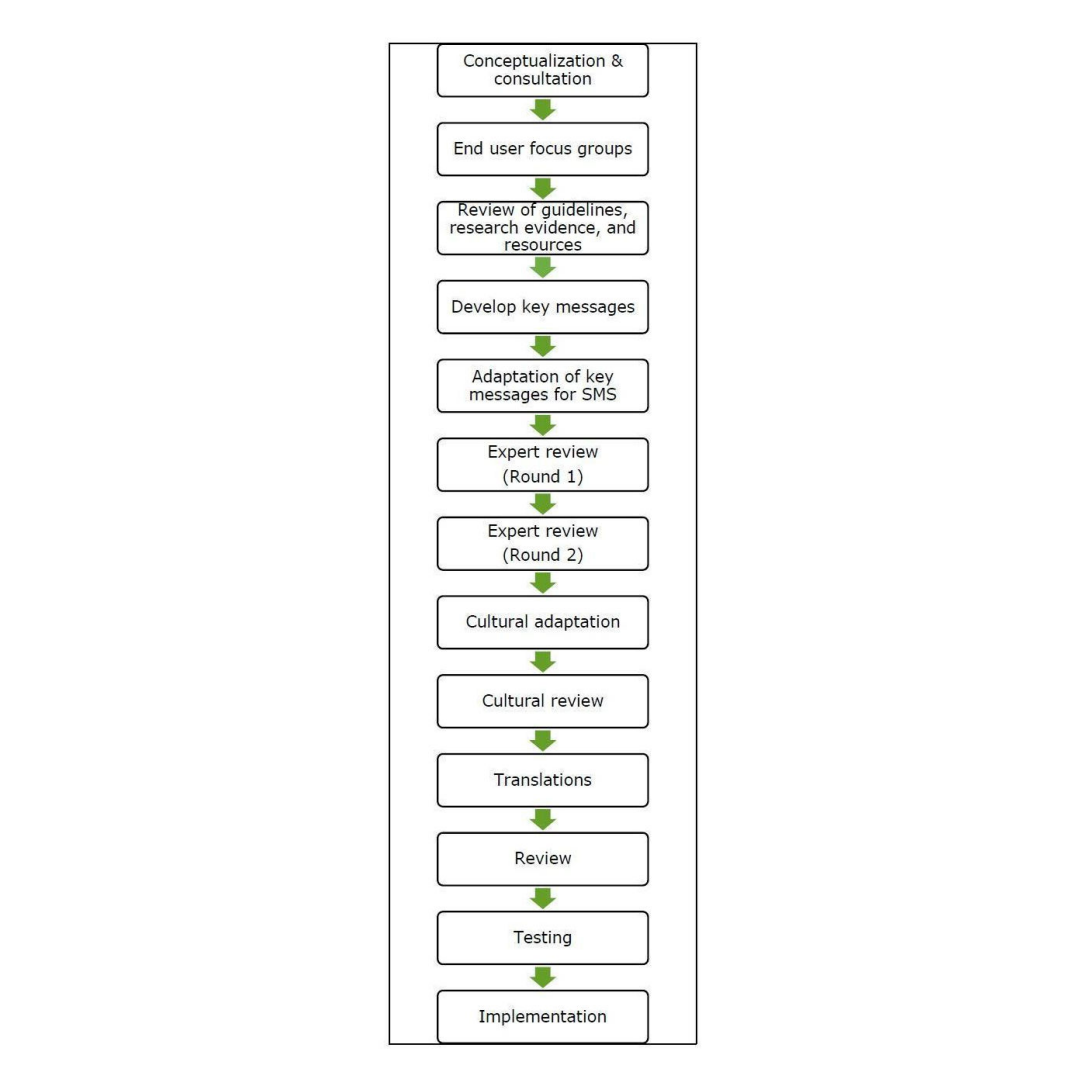
TextMATCH development process. SMS: short message service.

The development of TextMATCH was guided by our previously developed mHealth Development and Evaluation framework [24], which provides a process to direct the development and testing of mHealth interventions, with a focus on implementation from the beginning, use of behavioral change theory, and involvement of the target population. A summary of the development process can be seen in Figure 1.

A governance group for the broader HBHF initiative, the Roopu Kaitiaki, provided guidance on the primary focus and scope of the program. This group represented a consortium of community-based providers working with the target audience of the program. The target population was defined as pregnant women and mothers of babies aged under 2 years of Māori, Pacific, Asian, and South Asian ethnic communities within two health districts in Auckland, New Zealand. The target population also included their extended families (whānau in Māori) and anyone else who had influence on the health and wellbeing of the pregnant women, babies, mothers, and their preschool children.

The overarching principles agreed were

- education for those with low health literacy;
- targeting those who have relatively low involvement with conventional support networks;
- providing practical tips and suggestions that are relevant to their specific contexts;
- supporting improvements in health nutrition and uptake of appropriate physical activity;
- linking pregnant women or mothers of young children and their families to health and community services as early as possible; and
- using proven behavior change techniques and theories.

#### Formative Work

Formative work was conducted to inform development of the program including the design, content, and implementation. This included focus groups with the target audience and a review of the literature, guidelines, and publicly available resources.

The focus groups aimed to determine how the intended audience engaged with technology, how a technology-based health program could benefit them during pregnancy and the first 2 years of their child’s life, and to understand cultural norms, traditions, and beliefs around technology and maternal health. A total of 4 culture-specific focus groups were conducted with pregnant women, mothers of children aged under 2 years, and their support people. Participants were identified by the community organizations involved in the wider HBHF initiative. All groups were positive about using technology to support people to eat well and exercise during pregnancy and the first 2 years of a baby’s life. Technology preferences differed, with some groups reporting a preference for mobile phone messaging apps (such as WeChat, WhatsApp), whereas others reported nonconsistent mobile phone ownership and a preference for text messaging (short message service, SMS) due to limited data access. Other factors of importance for a mHealth program were being free, accessible on all types of mobile phones, and taking into consideration that phones are sometimes shared among family members. Therefore, in order to ensure greatest reach of the intervention, SMS was agreed on as the mode of delivery. Participants felt strongly that there was benefit to the messages being offered to partners or fathers and other family members, in addition to a pregnant woman or mother.

Consistent across the groups was a preference for New Zealand-based guidance and references, as there was concern that advice in internationally available apps may differ from that in New Zealand. Differences in terminology were identified, as well as culture-specific foods, practices, traditions, and activities, providing rationale for culturally adapted versions of the program. Preferences for languages varied, with all groups recommending an English option and some Asian participants requesting Simplified Chinese and Korean. In addition, consulted health care providers recommended adding Japanese and Te Reo Maori languages. Women in the Pacific Island and Indian focus groups predominately felt that an SMS intervention would be best in English. This was due to wanting the intervention to be in the language they were likely to be communicating with their lead maternal carers (LMCs) in for consistency, as well as a general preference for texting in English.

A review of the current literature, New Zealand guidelines, and resources was undertaken to establish key messages related to nutrition and physical activity for the target population, as well as other relevant areas.

#### Content Development

Findings from the formative work resulted in the development of key messages for the intervention. The key messages were then adapted into text messages limited to 160 characters. These text messages were sent to a technical advisory group (TAG, including local representatives from midwifery, public health, nutrition, physical activity, Māori health promotion, primary care, and postnatal providers) and revised before being sent to a second panel of experts (obstetrician, pediatrician, and academic experts in nutrition and physical activity) for review to ensure factual correctness. Once the key text messages were confirmed, they were then adapted into 4 cultural versions using the feedback from the formative work. These culturally adapted messages were reviewed and adapted further by the 4 cultural community groups in the consortium. Each cultural version was adapted into 2 versions (total 8 versions); one for mothers and the second one for other family members. Where needed, the messages were then translated into other languages (Te Reo Maori, Chinese, Korean, and Japanese) resulting in 16 different versions of the TextMATCH program (see Table 1 for a breakdown of the different versions). These were reviewed and back-translated by the 4 community groups. The messages underwent a final review before being loaded into the system and tested. Although the development process was linear (Figure 1), if, at later stages in the development process, changes to content were made, the affected content was rereviewed by all levels of reviewers before being translated, rereviewed, and tested.

Versions of the program that were delivered in English included greetings and other key words in the appropriate language (eg, Hindi, Samoan, Tongan, Cook Island Maori, Tuvaluan, and Tokelauan). The messages were also personally tailored by including the participant’s name, baby’s name, and gender-specific terms for messages after the baby was born (eg, he or him or his). Examples of TextMATCH messages can be seen in Table 2.

**Table 1:** TextMATCH versions.

Version number	Culture	Language	Relationship to baby
1	Māori	Te Reo	Mother
2	Māori	Te Reo	Other family member
3	Māori	English	Mother
4	Māori	English	Other family member
5	Pacific	English	Mother
6	Pacific	English	Other family member
7	Asian	Chinese	Mother
8	Asian	Chinese	Other family member
9	Asian	Korean	Mother
10	Asian	Korean	Other family member
11	Asian	Japanese	Mother
12	Asian	Japanese	Other family member
13	Asian	English	Mother
14	Asian	English	Other family member
15	South Asian	English	Mother
16	South Asian	English	Other family member

#### TextMATCH Program

**Figure 2:**
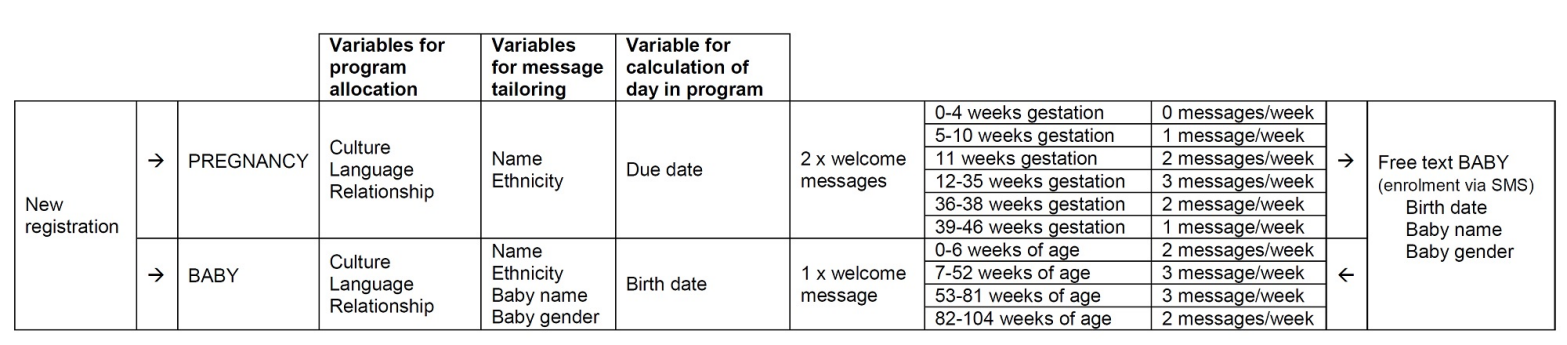
TextMATCH system overview. SMS: short message service.

The resulting TextMATCH program is individually tailored and available in 16 different cultural versions and 5 languages (Table 1). A person is referred to TextMATCH using a short referral form with questions to determine their specific TextMATCH program. Once registered, they receive up to 3 free text messages per week delivered to their mobile phone (see Figure 2). The estimated delivery date or baby’s birth date determines where in the TextMATCH program participants start. Messages are personalized (with the recipient’s name, the baby’s name, and gender) and tailored to the gestational age or age of the baby. Messages not only focus on nutrition and physical activity for pregnant women, baby, and wider family, but also cover a range of health topics including immunizations, safety, support services, weight gain, smoking, and emotional encouragement. The program is unidirectional, but if a person is registered during pregnancy they can self-switch to the baby messages, by free texting into the system when their baby is born. People can withdraw from the program by free texting the word “STOP” at any time. The program is provided at no cost to recipients including no cost to send text messages to switch to the baby program, or to withdraw from the program.

The TextMATCH system is automated following registration. Registration requires manual input of the recipient details for the generation of message schedules. In addition, the system is checked daily for unrecognized incoming messages and these are actioned if needed. The ongoing costs of running the program include the Gateway Company shortcode fees and per text message costs, as well as a maximum of 1 hour of person time per week to check the unrecognized incoming messages and manage issues.

**Table 2:** A selection of TextMATCH messages.

Example message	Timing	Culture	Relationship to baby
Malo e lelei [firstname]. You are now registered for TextMATCH. We will send you up to 3 messages per week to support you to eat well & be active while pregnant	Registration (during pregnancy)	Pacific-Tongan	Mother
Kia ora [firstname]. Exercise is important for your health & for the mums health during pregnancy, aim for 30 min of activity each day	Pregnancy (14 weeks)	Māori	Other family member
Iron is important to prevent you becoming too tired during pregnancy. Lean red meat, chicken, eggs, bajra flour, cowpea, masoor dal & moth beans contain iron	Pregnancy (23 weeks)	South Asian	Mother
Talofa [firstname]. Keeping active towards the end of pregnancy can be tough but the health benefits make it worth it. Try swimming or a very gentle walk	Pregnancy (36 weeks)	Pacific-Samoan	Mother
Hi [firstname]. Congratulations on the birth of [babyname]. We hope you are enjoying this special & exciting time. Thanks for having TextMATCH on board	Completion of switch to baby program	Asian	Mother
Kia ora [firstname]. Breast milk is the perfect kai for your baby - it’s healthy & it’s free! Please talk to your midwife if you need help or support	Postnatal (2 weeks)	Māori	Mother
Kia ora. Sounds, smiles, laughter, touch & interaction all help [babyname] to develop. Spending time with [him/her] will help to grow your relationship with [him/her]	Postnatal (17 weeks)	Māori	Other family member
Don’t forget to take care of yourself, this includes eating well & getting rest. To be able to look after [babyname] you need to take care of yourself first	Postnatal (22 weeks)	Pacific	Mother
Aim to feed [babyname] a variety of healthy choices in every meal. Try boiling pumpkin or kumara together with spinach or taro leaves for a nutritious option	Postnatal (44 weeks)	Pacific	Other family member
Choose healthy foods for [babyname]. There is no need to add salt, sugar, butter or ghee to [his/her] food. If you want to cancel these messages, reply text STOP	Postnatal (1 year 20 weeks)	South Asian	Mother

### Engagement and Acceptability of TextMATCH

TextMATCH went live in August 2014 with people referred to the program by community organizations within two urban health districts in New Zealand. We conducted a study, 18 months post go-live, to assess the engagement and acceptability of the program. The study had two aims:

1. To assess the engagement of participants with the TextMATCH program, including enrollment and disengagement rates.
2. To assess the acceptability of the TextMATCH program, including its cultural appropriateness and relevance, reasons for withdrawal, and to determine ways in which the program could be improved.

## Methods

### Engagement

Engagement data collected by the content management system was extracted on April 7, 2016. All data was kept private and secure to respect the privacy of all intervention participants. Data collected for the first 18 months was used in this analysis. The data for each participant included program number (indicating cultural group, language, and relationship); month registered; due date month and year; baby birth date and year; whether they switched to the baby program from the pregnancy program; whether they texted “STOP;” their withdrawal date (if applicable); and completion date (if applicable). Descriptive statistics were generated for baseline demographic and clinical characteristics, and measures of engagement with the system. Counts and percentages were reported for categorical variables, along with means and standard deviations for continuous variables.

### Acceptability

Phone interviews were conducted between January and March 2016. All study documents and procedures were approved by the New Zealand Health and Disability Ethics Committee (15/CEN/210).

Participants included those who were registered for the TextMATCH program between August 2014 and August 2015, and who had previously agreed to be contacted about their experience with the program. Potential participants were excluded if it was known that they had experienced a miscarriage; their due date fell during the study period; they only spoke Japanese or Korean, as no interviewer was available for these languages. We also excluded those who withdrew from the program by texting “STOP” after receiving less than 2 weeks of messages, as it was determined that this group would not have received sufficient intervention to provide feedback for this study. To ensure participants with a range of experiences with the program were interviewed, potential participants were split into four categories:

1. those who had received TextMATCH for 6 months or more;
2. those who had received TextMATCH for 3-6 months;
3. those who withdrew from the program by texting “STOP” after receiving a minimum of 2 weeks of messages;
4. those that received TextMATCH during pregnancy but did not self-switch to the baby messages following their baby’s birth.

Eligible participants were sent a text message asking if they would be interested in being contacted for an interview. In addition, community providers contacted some eligible participants to invite them to participate, to accommodate those who did not have sufficient credit on their phones to respond, and to ensure adequate numbers. If participants replied “yes,” they were called by the research assistant for the interview. Before commencing with the interview, they read the participant information sheet and consent form, and verbal consent was then obtained. Interviews were conducted in English or Chinese based on participant preference. Following informed consent, the researcher then went through the interview schedule with participants covering the following topics:

- General feedback
- Usefulness of the messages
- Appropriateness of the messages, with special attention to the cultural adaptation of messages
- Perceived impacts
- If withdrew: reasons for withdrawal
- If did not self-switch: reasons for not choosing to switch to baby messages
- Suggestions for improvements

The interview data was analyzed and summarized using descriptive quantitative analyses including means, standard deviation and proportions, and qualitative comments analyzed using simple thematic analysis.

## Results

### Engagement

In the first 18 months of the TextMATCH program, 1404 individuals enrolled and over 98,000 messages were sent by the system. The percentage of participants enrolled in each of the 16 different versions of TextMATCH varied (Table 3). Even though TextMATCH is offered to other family members, 72.44% (1017) of individuals enrolled were mothers—almost three times more than other family members.

**Table 3:** Enrollment in TextMATCH by version.

Version	Cultural group	Language	Relationship to baby	n (%)
1	Māori	Te Reo	Mother	17 (1.21)
2	Māori	Te Reo	Other family member	8 (0.57)
3	Māori	English	Mother	173 (12.32)
4	Māori	English	Other family member	55 (3.92)
5	Pacific	English	Mother	244 (17.38)
6	Pacific	English	Other family member	39 (2.78)
7	Asian	Chinese	Mother	283 (20.16)
8	Asian	Chinese	Other family member	67 (4.77)
9	Asian	Korean	Mother	51 (3.63)
10	Asian	Korean	Other family member	22 (1.57)
11	Asian	Japanese	Mother	8 (0.57)
12	Asian	Japanese	Other family member	0 (0.00)
13	Asian	English	Mother	19 (1.35)
14	Asian	English	Other family member	14 (1.00)
15	South Asian	English	Mother	222 (15.81)
16	South Asian	English	Other family member	182 (12.96)

Of the 1404 individuals who enrolled in TextMATCH, 260 (18.52%) actively discontinued the program by texting the word “STOP.” Discontinuation rates varied between the TextMATCH cultural versions; 14.1% of those registered for the Asian versions of the program (#7-14) texted “STOP,” 15.9% of those registered for South Asian versions (#15 and 16), 19.9% of those registered for Pacific versions (#5 and 6), and 29.2% of those registered for Māori versions (#1-4).

For those who texted “STOP,” the average duration of messages received was 3.84 months (SD 3.10; range 0-17 months). Of the 502 individuals who were sent text messages prompting them to switch from the pregnancy version of the program to the baby version, 356 (70.9%) fully completed the three text messages required to switch to the baby program—74.6% of mothers and 59.1% of other family members.

### Acceptability

A total of 52 people agreed to be contacted for an interview. Of these, 5 declined to participate, 1 was unable to provide consent, and 17 were unable to be contacted. A total of 29 interviews were completed. Of those who participated, 12 (41%) had received TextMATCH for 6 months or longer, 9 (31%) had received TextMATCH for 3-6 months, 6 (21%) had withdrawn from TextMATCH by texting “STOP,” and 2 (7%) had not switched from pregnancy to baby messages.

The mean number of months participants had received TextMATCH messages for was found to be 7 months (range 3-16). Those who had stopped the messages or did not switch over (n=8) received on average 6 months (range 3-9) of messages. A summary of the TextMATCH version that the interview participants had received can be seen in Table 4, and participant’s demographic characteristics in Table 5.

**Table 4:** Interview participants by TextMATCH version.

TextMATCH^a^ program #	Description (cultural group, language, relationship)	No. of participants	No. by type of messages received		
			Only pregnancy messages	Only baby messages	Both pregnancy and baby messages
3	Māori, English, mother	4		2	2
5	Pacific, English, mother	5	1	3	1
7	Asian, Chinese, mother	11	3		8
8	Asian, Chinese, other family member	2	1		1
15	South Asian, English, mother	6			6
16	South Asian, English, other family member	1		1	

All but 2 participants (27; 93%) reported that they found the messages personally useful. For those who found it useful, the mean rating on a scale of 1 (a little useful) to 5 (extremely useful) was 4.24 (SD 0.80; range 2-5). Table 6 shows the mean ratings by interview category and TextMATCH cultural version. Many participants commented generally on the usefulness of messages including that TextMATCH provided a timely reminder of information which they previously knew. Participants reported that the messages were also reassuring, encouraging, and motivating. The 2 participants who reported that the messages were not useful had reported finding the content in the messages too basic and therefore not beneficial to them.

**Table 5:** Characteristics of interview participants (n=29).

Characteristics			Number, n (%)
**Gender: Female**			26 (90)
**Relationship**			
	Mothers	26 (90)	
	Other family member(fathers)	3 (10)	
**Ethnicity**			
	Māori	5 (17)	
	Samoan	2 (7)	
	Niuean	1 (3)	
	Tuvaluan	1 (3)	
	Chinese	13 (45)	
	Indian	4 (14)	
	Nepalese	2 (7)	
	Sri Lankan	1 (3)	
**Mean age (SD^b^** **)**			32.6 years (range 23–44)

^a^TextMATCH: Text for MATernal and Child Health.

^b^SD: standard deviation.

**Table 6:** Mean ratings of usefulness for those reporting the program to be useful (n=27).

Grouping		n	Mean rating (SD^a^)	Range
**Interview category**				
	6+ months	11	4.32 (0.64)	3-5
	3-6 months	9	4.67 (0.50)	4-5
	Withdrew	5	3.20 (0.84)	2-4
	Did not switch	2	4.50 (0.71)	4-5
**TextMATCH^b^** **version**				
	Māori (#1-4)	4	4.00 (0.82)	3-5
	Pacific (#5-6)	5	4.20 (0.84)	3-5
	Asian (#7-14)	11	4.23 (0.61)	3-5
	South Asian (#15-16)	7	4.43 (1.13)	2-5

^a^SD: standard deviation.

^b^TextMATCH: Text for MATernal and Child Health.

All participants responded that the messages were relevant to them, with many commenting that some were more relevant than others (28; 97%). In addition, all study participants reported finding the messages culturally appropriate and easy to understand (29; 100%). Participants provided positive feedback on the personalization of the messages; they enjoyed that the messages included greetings in languages other than English and having their names in the messages.

When asked whether they found the specific food practices and activities mentioned in messages appropriate to them, all but three reported that they were (27; 93%). Reasons why food practices and activities were less relevant to participants included that messages referring to meat were not relevant for vegetarians, and that messages referring to dairy products or to prepreparing and freezing meals were not relevant for many Chinese people.

**Table 7:** Perceived impacts of TextMATCH.

Impact	Number responded	Response YES: n (%)
Improvements to eating habits	29	18 (62%)
Improvements to family’s eating habits	29	16 (55%)
Positive changes to food shopping	29	19 (66%)
Positive impact on exercising	29	15 (52%)
Improvements to knowledge or understanding	29	21 (72%)
Feeling more supported	28	28 (100%)

Participants were also asked if there were any messages that they found irrelevant or inappropriate with only 6 (21%) reporting that there were. Of these, 3 felt that some proportion of messages did not apply to their own pregnancy or child, but many also commented that they were happy to ignore these. Only one respondent felt that these represented a significant proportion of the program, and said that this was because she had a complicated pregnancy and birth.

When asked if they would have preferred the messages to be in a different language, most (25; 89%) responded “no.” One participant would have preferred the messages in Hindi, another in Samoan, and one was happy with her Māori cultural messages in the English language but would have preferred more phrases and words in the Te Reo language. A separate question sought participants’ suggestions about the provision of messages in other languages. Suggested languages included Samoan (n=5), Hindi (n=5), Tongan (n=1), Niuean (n=1), and Spanish (n=1). Some of those suggesting Pacific languages stated that this would be useful to improve their own Pacific language competency rather than assisting people who did not speak English.

#### Perceived Impacts

Participants were asked about the impacts that they felt TextMATCH had on themselves, their family, and their baby (see Table 7). A common benefit of the messages appeared to be the reminders and reassurance they provided:

(when it’s) a stressful time and then you get the message and realize you're not doing too bad.

Pacific, mother

Over 50% of participants reported that the program had improved both their eating habits (18; 62%) and exercise (15; 52%). Furthermore, all participants reported feeling supported by messages (28; 100%). Those who felt they had not gained knowledge from the program still found it useful because it made them feel more supported. One mother commented:

there’s a lot of time in isolation when it’s just you and bubba, it’s quite nice to receive that message.

Māori, mother

A total of 21 (72%) participants reported improvements to their knowledge and understanding. Some participants felt that the program was ideally suited for first-time parents with less baseline knowledge. However, one user stated that:

it’s quite empowering actually just to get that little bit more information you think you know everything after seven children so it’s actually quite cool.

Māori, mother

A total of 12 (41%) participants reported that they had family members also receiving the messages. All remarked that it was good for the partners to receive messages, as it put both parents “on the same page” with regard to pregnancy and child care, and many discussed message content. Many mothers also felt that their partners were better able to care for them and the baby as a result of the messages.

All participants (29; 100%) reported that they would recommend the TextMATCH program to others. Reasons included that it provides useful information and tips, uses a medium which is very accessible and unobtrusive, provides valuable reminders, and can benefit the whole family. Respondents also mentioned that messages provided valuable support and encouragement and would be especially useful for first time and younger mothers.

#### Reasons for Disengagement

There were 6 participants (21%) who had texted in “STOP” to withdraw from the program after an average duration of 6 months of messages (range 4-9 months). Four of these participants reported that they withdrew due to not finding the messages very useful or beneficial, one due to a technical issue, and one reported that they had only wanted the messages during pregnancy and so stopped once their baby was born. There were 2 participants (7%) who had not chosen to switch to the baby messages after completing the pregnancy messages. One reported that they had not realized that they had to text back to sign-up for the baby messages, and the other reported that things were hectic after the baby was born and so they forgot to switch.

#### Suggestions for Improvements

A total of 20 participants provided suggestions for how the program could be improved. Most commonly, participants suggested that the messages could contain more detailed and specific information on the baby’s diet (especially solid foods), recipes, and activities (like where to play outdoors; n=13). Others thought that bidirectional messages to be able to ask about certain aspects of the program would be useful (n=5). Two participants suggested having extra optional message streams for certain maternal health conditions (eg, hypertension or diabetes), areas of interest (eg, diet), or for multiple pregnancies (eg, twins). Other suggestions included information about local events (n=1), the use of other social media like WeChat to enable photo or video messages (n=1), more emotional support for mothers (n=1), changes to the frequency or timing of messages (n=4), and better channels for users to recommend the program to others (n=1).

## Discussion

### Principal Findings

The process of developing TextMATCH with specific cultural, family, and language versions was extensive and resource-intensive. It involved focus groups and testing directly with the target audience. It also involved considerable input from the community groups working with those families. This resulted in what appears to be a rather simple program to the end-user, but which is actually composed of 16 different versions. The system rules ensuring that the correct version is sent to each participant are based on just a small number of questions. The engagement statistics and feedback from participant interviews have indicated that there is value in this process, with positive responses to the personalization and relevance of the culturally tailored messages. Other benefits of our program include the feelings of support reported by all participants, and the inclusion of other family members receiving the messages.

Some participants reported that some of the messages were not useful for them, inappropriate or irrelevant. This feedback has been taken into consideration and the program is being improved based on their suggestions. Regardless, all those interviewed, even those reporting it not to be of personal use, reported that they would recommend the program to others. Although the program appears to be well-received and perceived to be of benefit, evaluation of its effectiveness has yet to be undertaken and will be necessary to understand if it is impacting on health outcomes.

Feedback from recipients as well as changes in evidence and guidelines mean changes to the content of programs such as TextMATCH need to be made from time to time. In this case, messages are updated using the same process, requiring significant time and input from reviewers before they are loaded into the content management system. This needs to be factored into the ongoing costs of the program.

We believe that it is important that mHealth programs target minority and vulnerable populations, who may have difficulty accessing traditional health services due to a variety of reasons. These reasons can include language, cultural differences, geography, travel, work, or roles caring for others. It is also important to consider the technological infrastructure of a setting, and the power dynamics which determine different groups’ access to technologies, and how existing communities and health systems will respond to mHealth [3,7,25].

### Limitations

It is important that these findings be interpreted in light of the limitations including the small number of participants interviewed, potential sampling bias in those who agreed to an interview, and unequal samples of cultural groups and interview categories. The overall numbers of people using the Korean or Japanese versions of the program at the time of interviewing were not large and interviews could not be offered in these languages, meaning the results cannot be generalized to those receiving TextMATCH messages in those languages. Further investigation is needed into reasons for disengagement in those who had withdrawn from TextMATCH within 2 weeks of signing up as this group was not included in the study and could provide valuable insight into engagement.

### Conclusions

We have described how mHealth can provide access to health information, motivation, reassurance, and support in simple programs like TextMATCH. It can also link people to existing community programs for appropriate in-person support. It is important that mHealth programs are designed with the involvement of these vulnerable groups in our populations, and that we take the time and extra resources required to ensure that the programs will be relevant and appropriate for the context of their daily lives. This is not simply about translating programs that have been developed for the majority. We have described here an extensive process of developing an intervention with minority populations first. There is no “majority” (pakeha /New Zealand European) version of TextMATCH. This process has begun in collaboration with the community which cares for these families of minority cultural groups and which shares their culture.

## Abbreviations

HBHF: Healthy Babies Healthy Futures
HIV: human immunodeficiency virus
LMC: lead maternal carer
MAMA: Mobile Alliance for Maternal Action
mHealth: mobile health
SD: standard deviation
SMS: short message service
TAG: technical advisory group
TextMATCH: Text for MATernal and Child Health
